# Outer membrane vesicles: A bacterial-derived vaccination system

**DOI:** 10.3389/fmicb.2022.1029146

**Published:** 2022-12-21

**Authors:** Linda A. Lieberman

**Affiliations:** Discovery Immunology, Merck Research Laboratories, Merck & Co., Inc., Cambridge, MA, United States

**Keywords:** vaccine, bacteria, outer membrane vesicle, lipopolysaccharide, glycoengineering

## Abstract

Outer membrane vesicles (OMVs) are non-living spherical nanostructures that derive from the cell envelope of Gram-negative bacteria. OMVs are important in bacterial pathogenesis, cell-to-cell communication, horizontal gene transfer, quorum sensing, and in maintaining bacterial fitness. These structures can be modified to express antigens of interest using glycoengineering and genetic or chemical modification. The resulting OMVs can be used to immunize individuals against the expressed homo- or heterologous antigens. Additionally, cargo can be loaded into OMVs and they could be used as a drug delivery system. OMVs are inherently immunogenic due to proteins and glycans found on Gram negative bacterial outer membranes. This review focuses on OMV manipulation to increase vesiculation and decrease antigenicity, their utility as vaccines, and novel engineering approaches to extend their application.

## Introduction

**FIGURE 1:**
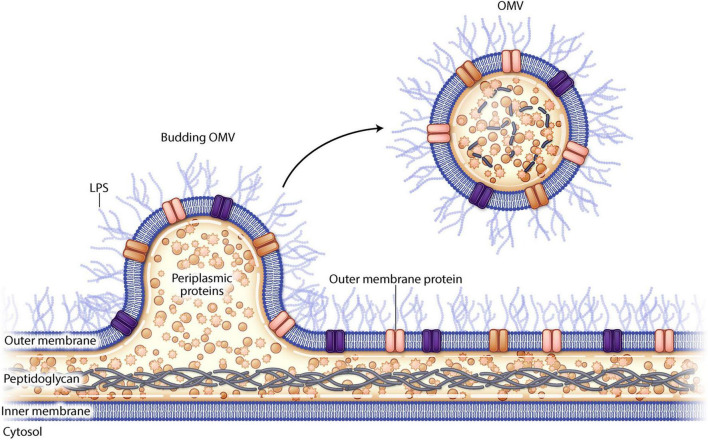
Schematic drawing of outer membrane vesiculation from a gram negative bacterial cell wall.

Outer membrane vesicles (OMVs) are small spherical lipid nanoparticles (LNP) (20–300 nm) that derive from the outer membrane of gram-negative bacteria (Figure 1). They resemble the outer membrane of the bacterial strain from which they are derived and are composed of bacterial proteins, lipids, nucleic acids, and periplasmic contents. OMVs from pathogenic bacteria can fuse with other bacterial membranes delivering virulence factors such as toxins and antibiotic resistance plasmids (Collins and Brown, 2021). There is intrinsic variability between OMVs derived from the various bacterial species which can be attributed to differences in cell envelope composition and differential bacterial gene expression (Schwechheimer and Kuehn, 2015). OMVs can be released by either pathogenic or non-pathogenic organisms and though the OMV can cause disease pathogenesis, it cannot cause disease on its own as it is non-replicative.

Vesiculation and OMV composition is influenced by temperature, growth environment, quorum sensing, and growth phase of the bacteria (Cecil et al., 2019). Many Gram-negative bacteria naturally release OMVs during the growth process or due to environmental stress (e.g., nutrient deprivation, antibiotic exposure, and oxidation) which contributes to enhanced bacterial fitness. They can integrate into biofilms promoting biofilm formation by contributing necessary nutrients (Micoli and MacLennan, 2020). It was once believed that only gram negative bacteria can produce OMVs, yet it has recently been reported that some gram positive bacteria can produce extracellular vesicles akin to OMVs, though these are less well characterized (Liu et al., 2018).

OMVs can modulate host immunity due to the immunogenic proteins and glycans found on their surface, lending them natural adjuvant properties and suggesting OMVs could be used as a vaccination platform. It has been demonstrated that the intrinsic adjuvant activity elicits both innate and adaptive immune response (Tan et al., 2018). A potential liability of using OMVs for vaccination is that the immunogenicity could cause toxic shock, but this may be overcome by modulating lipopolysaccharide (LPS) levels on the OMV surface using genetic manipulation, or detergents for extraction of OMVs from bacterial cells. Another challenge with OMVs as a vaccine platform is that the protein expression on the cell surface may be inconsistent leading to batch-to-batch variation.

The obvious approach for OMV based vaccination is to use homologous bacterial strains to vaccinate individuals against the pathogen from which the OMVs derive. Yet advances in genetic engineering and glycobiology allow OMVs to be manipulated to display heterologous proteins from other bacteria, viruses, and parasites. Engineered OMVs can even be used to display tumor antigens (Balhuizen et al., 2021). Additionally, there are efforts to use OMVs as drug delivery systems by loading them with specific cargo, and this has been incorporated into therapeutic vaccine design (Gujrati et al., 2014).

## Isolation of OMVs

Outer membrane vesicles occur spontaneously in nature in response to stress conditions. These spontaneous OMVs (sOMVs) are the most similar to the native state of the bacterial membrane from which they derive, making them ideal for homogenous immunization. Though it should be noted sOMVs are naturally released in low quantities questioning their utility as a vaccine platform. The amount of outer membrane/periplasmic proteins packaged into OMVs varies by organism. It has been reported that *Escherichia coli* may package as little as 0.2% of these proteins into OMVs whereas *Neisseria meningitidis* can incorporate as much as 12% of its protein into OMVs (Li and Liu, 2020).

From a manufacturing perspective, yields must be increased to make OMVs a viable tool. Different approaches have been taken to increase OMV vesiculation and their advantages and disadvantages are outlined below.

### OMVs derived from mechanical disruption

Mechanical disruption of bacterial membranes using physical (non-detergent) disruption, such as extraction with EDTA, sonication, or vortexing, can result in an increased yield of OMVs (Gnopo et al., 2017). LPS molecules are negatively charged and calcium molecules in the membrane keep the LPS from repelling itself. Using a calcium chelator for isolation (such as EDTA) destabilizes the bacterial membrane resulting in OMV release. This approach yields OMVs that more closely resemble the native bacterial membrane and they elicit comparable immune responses (Gnopo et al., 2017). Sonication of bacterial pellets fragments the whole bacterium and these fragments fuse together to form OMVs. While this increases yields, it also leads to a higher occurrence of non-membrane components being included in the OMVs (Balhuizen et al., 2021); vortexing the bacteria would likely have a similar effect. The non-membrane components introduced into the OMV using these methods may increase antigenicity, but concurrently decrease safety (Gnopo et al., 2017).

### Detergent extraction

One of the earliest adopted techniques to improve the yield of OMVs is the use of detergent applied to intact bacteria. Detergents such as deoxycholate or sodium dodecyl sulfate are commonly used and the resulting OMVs have decreased levels of LPS thereby reducing toxicity of the OMVs (van der Pol et al., 2015). A drawback to using detergent extraction is that this process also results in the loss of many bacterial antigens and lipoproteins which decreases the inherent adjuvant effect of OMVs (Balhuizen et al., 2021). Therefore, adjuvants must be added to these OMVs to elicit a productive immune response. Additionally, detergent extracted OMVs are more likely to aggregate making purification challenging.

### Genetically manipulated OMVs (gOMVs)

**FIGURE 2:**
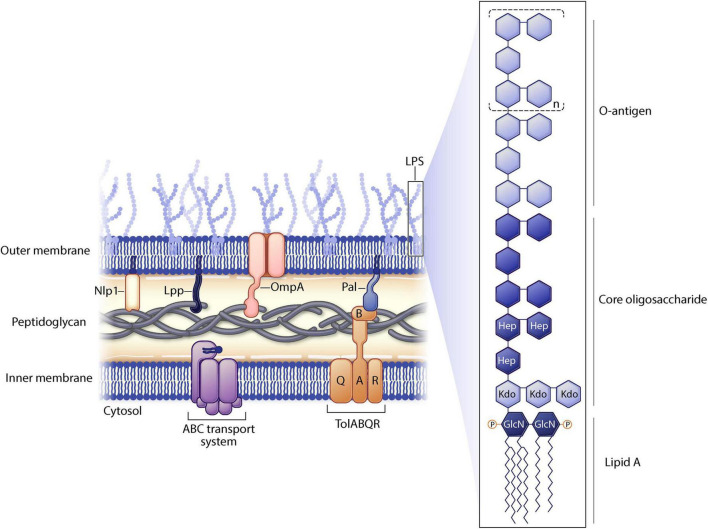
Gram negative cell membrane structure. Selected proteins manipulated in outer membrane vesicle (OMV) engineering are shown. Lipopolysaccharide (LPS) substructures are highlighted.

Genetic manipulation of bacterial genes can enhance vesiculation thereby resulting in increased yields of genetically manipulated OMVs (gOMVs). Often these genetic disruptions destabilize linkages to the outer membrane. As a reminder, the gram-negative envelope consists of two lipid membranes (outer and inner) sandwiching a peptidoglycan layer. There are lipoproteins, such as Lpp and NlpI, and various outer membrane proteins such as OmpA located in the bacterial envelope (Figure 2). LPS (found only on gram negative bacteria) is a highly immunogenic outer membrane structure and it can be modified to decrease OMV toxicity.

These gOMVs have been genetically manipulated disrupting various bacterial genes, though the most common is the disruption of genes that belong to the Tol-Pal system, which are involved in linking the membranes to the peptidoglycan layer (Bernadac et al., 1998). The Tol-Pal complex is involved in cell division, forming at the septal ring during cytokinesis (Park and Cho, 2022). Tol-Pal mutants can induce release of periplasmic proteins, perturbing outer membrane integrity. For instance, a Δ*tolR* mutant of *Shigella flexneri* results in 8× greater OMV production as compared to a wild type strain (Pastor et al., 2018). Even more impressively, a TolA mutant strain of *Shigella boydii* increases OMV formation by 60% (Mitra et al., 2016). These are just a few examples of how genetic manipulation of this pathway can increase vesiculation. However, it should be noted that the Tol-Pal system is not found in all gram-negative species.

Other proteins found in gram negative bacteria can be manipulated to increase vesiculation. NlpI is an outer membrane protein involved in cell division and deletion of *nlpI* from *E. coli* results in disrupted peptidoglycan crosslinking which destabilizes the outer membrane, leading to hypervesiculation (Schwechheimer et al., 2015). Additionally, Lpp is a major lipoprotein covalently linked to the peptidoglycan layer on its C terminus while the N terminus of Lpp is inserted into the outer membrane layer, maintaining the integrity of the membrane. Defects in Lpp have been shown to result in hypervesiculation of *E. coli* and *Yersinia pestis* species (Micoli and MacLennan, 2020). OmpA mutations lead to OMV overproduction in *Acinetobacter baumannii*, *Vibrio cholerae*, *E. coli*, *Salmonella* spp., and other strains due to changes in the outer membrane structure, likely causing decreased crosslinking between the outer membrane and peptidoglycan (Schwechheimer and Kuehn, 2015; Avila-Calderón et al., 2021). Furthermore, the ATP-binding cassette (ABC) transport system has been disrupted in *Haemophilus influenzae*, *V. cholerae*, and *E. coli* leading to accumulation of phospholipids in the outer membrane, thereby increasing vesiculation (Micoli and MacLennan, 2020). Additional genes have been identified in different gram-negative bacterial strains that lead to similar disruptions and increased OMV yields.

## Heterologous OMVs

Thus far, OMVs expressing native homologous proteins have been discussed. It is not always possible to express antigenic proteins of interest in OMVs from the parental bacterial strain as some strains of gram-negative bacteria are more challenging to grow in the laboratory due to stringent growth requirements, slow growth rate, or low vesiculation rates making yields low and challenging to manufacture. In this case, it may be possible to express the desired proteins in a heterologous vesiculating bacterial strain. There are certain bacterial species, like *E. coli*, that are more permissive to genetic manipulation and produce OMVs at a higher yield allowing them to be used as a universal delivery system. Using non-pathogenic bacterial strains to express these heterologous antigens can reduce the toxicity, while the pathogen-associated molecular pattern molecules (PAMPs) present on these strains still induce an immune response against the expressed antigen (Gnopo et al., 2017). Elegant genetic solutions are summarized below, but it should be noted that an alternative approach is to attach heterologous polysaccharides (or proteins) using conjugation chemistry to link the antigen directly to the outer membrane of the OMV (Di Benedetto et al., 2021).

### Recombinant OMVs

One commonly used approach to express a heterologous protein on the surface of a bacterial strain is by fusing a target protein to a membrane associated protein such as the carrier protein cytolysin A (ClyA), a transmembrane protein enriched on secreted OMVs. A protein antigen gene can be fused to the *clyA* gene and the plasmid is introduced into the bacterial strain which will then express the desired antigen on the OMV surface. Since ClyA is itself immunogenic, it will enhance the immune response to the antigen (Gnopo et al., 2017; Zhu et al., 2021). An example of this approach was the fusion of the outer membrane protein (Omp22) of the medically important bacterium *A. baumannii*, with ClyA in the *E. coli* DH5α strain. Mice were immunized with these OMVs and challenged with *A. baumannii* and increased survival was observed (Huang et al., 2016). As in the example above, the fused antigen can be of bacterial origin, but it need not be. The non-pathogenic *E. coli* strain Nissle 1917 has been manipulated to express a fusion protein of ClyA with the (viral) influenza antigen M2e4×Het. Mice were immunized with OMVs loaded with this fusion protein and then challenged with a lethal dose of influenza; all of the immunized animals survived whereas unvaccinated animals succumbed 10 days post infection (Rappazzo et al., 2016).

### Glycosylated OMVs (glyOMVs)

Another approach to express heterologous proteins on OMVs is through glycoengineering of the LPS O antigen. LPS consists of three portions (Collins and Brown, 2021): the outermost portion of LPS is the O antigen, consisting of polysaccharides (Schwechheimer and Kuehn, 2015); the central portion of LPS is the core oligosaccharide (Cecil et al., 2019); and lipid A is the inner most portion of LPS which is highly conserved and is found inserted into the outer membrane (Figure 2). The O antigen is highly variable between species in composition, length, and linkages. Furthermore, within a species, O antigens can differ across different serovars increasing virulence of different bacterial strains. The gene for the O antigen from a pathogen can be expressed in a non-pathogenic O-antigen mutant strain of bacteria. Recombinant O antigen polysaccharide biosynthesis is coupled with vesiculation in non-pathogenic strains of *E. coli* resulting in glycosylated OMVs (glyOMVs). The heterologous O antigens are synthesized and attached to the lipid A-core of the *E. coli* strain (*via* the endogenous ligase) and the heterologous O glycan is expressed on the outer membrane (Gnopo et al., 2017).

One group described using a hypervesiculating *E. coli* strain (*tolRA* knockout) to express O antigens from various pathogens, setting up a plug-in platform (Chen et al., 2010). They expanded studies on one construct that expressed the O polysaccharide of *Francisella tularensis* subsp. *tularensis*, a highly virulent bacterial strain. They used the *E. coli* glyOMV expressing the *F. tularensis* O antigen to vaccinate BALB/c mice which were subsequently challenged with *F. tularensis*. Significant levels of IgG and IgA antibodies specific for this antigen were detected and mice displayed a delayed time to death following lethal challenge (Chen et al., 2010). Similarly, another group successfully bioconjugated polysaccharides from two hypervirulent strains of *Klebsiella pneumoniae* into a glycoengineered *E. coli* strain resulting in protective effects upon vaccination and challenge (Feldman et al., 2019). These are just a few examples of the creative approaches some groups have begun to take to build recombinant OMVs. It should be noted that the variability of O antigens across certain bacterial species could limit the broad applicability of this vaccination approach.

## Immune response to OMVs

Outer membrane vesicles are the right size for immune cell uptake and the fact that they can express bacterial surface antigens in their native conformation make them an ideal delivery system for bacterial proteins (Mancini et al., 2020). The outer membrane of the OMV contains PAMPs (largely conserved across microbes) which activate pattern recognition receptors (PRRs) on the host cells (immune and non-immune), and this activates various immune pathways. Toll-like receptors (TLRs) are well characterized PRRs and multiple TLRs on the host cell membrane are activated by OMVs. It has been demonstrated that OMVs can induce both cellular and humoral memory responses following vaccination (Bottero et al., 2016; Zurita et al., 2019).

Outer membrane vesicles contain intrinsic adjuvant properties due to the presence of PAMPs that act as TLR activators, such as LPS, flagellin, lipoproteins, and peptidoglycans (Tan et al., 2018). TLR4 recognizes the lipid A protein of LPS, and TLR2 dimerizes with TLR1 to recognize bacterial lipoproteins. TLR5 recognizes flagellin which is a protein found in OMVs of flagellated bacterium, and intracellular TLR9 recognizes unmethylated CpGs which are present in prokaryotic genomic DNA (Mancini et al., 2020). These interactions can induce proinflammatory cytokine release. In addition, innate immune cells recognize and phagocytose OMVs leading to an adaptive immune response triggered by antigen presentation, resulting in activation of both CD4^+^ and CD8^+^ T cells.

The antigenic nature of native LPS can be either positive or negative for this delivery system. LPS O antigens vary greatly (e.g., sugar composition and length) across bacterial strains and this will affect the toxicity of the OMV. LPS acts as an adjuvant which will effectively elicit immune response to the antigens being presented on the OMV surface, but too much LPS can be overly immunogenic and cause overstimulation of the immune system leading to systemic toxic shock. Modification of LPS lipid A (Figure 2), the portion of LPS that elicits TLR4/MD-2 signaling, can decrease the toxicity of LPS. Bacterial enzymes naturally modify lipid A structures in response to environmental stress or to evade host immunity (Raetz et al., 2007). In order to render OMVs less immunogenic, the acyl chains of lipid A can be decreased in length. It is known that lipid A molecules with fewer than six acyl chains induce a weaker immune response as compared to lipid A that is greatly acylated (Mancini et al., 2020). For instance, mutating lpxL1 in *N. meningitidis* yields the same amount of LPS as in wild type bacteria, but the lipid A is penta-acylated thereby decreasing activation of TLR4/MD-2 signaling and reducing LPS endotoxicity (van der Ley and van den Dobbelsteen, 2011).

As outlined above, OMVs expressing less reactive LPS are preferable, and this can be achieved using detergent preparation of OMVs or through genetic modifications. Depletion or modification of the msbB gene of *Salmonella typhimurium* or *E. coli* inactivates the encoded lipid A acyltransferase which results in OMVs with low endotoxin (Kim et al., 2009; Lee et al., 2009). Genetic modification of other genes in various bacterial strains have been carried out to the same outcome of decreased toxicity. Modification of LPS may decrease toxicity, but it also decreases the adjuvant properties of the OMV. Therefore, OMV vaccines that have been genetically modified to change the LPS structure likely will need adjuvants added to elicit a more substantial immune response and induce long term immunity.

It should be noted that alternative approaches to decrease toxicity of OMVs may be possible. It has been reported that pre-treating mice with all-trans retinoic acid (ATRA) prior to administration of *V. cholerae* OMVs decreased toxicity by downregulating TLR2 responses (Sinha et al., 2017). This can be attributed to the reported anti-inflammatory properties of ATRA. Furthermore, delivery of ATRA increased mucosal immune responses in this model. This can be explained as it has been described that ATRA induces α4β7 and CCR9 expression on T cells which facilitates lymphocyte migration and enhances immune responses (Tan et al., 2011). This example suggests that combining OMVs with other molecules (such as ATRA) can modulate toxicity and potentially provide an adjuvating effect.

### Routes of administration and immunity

Outer membrane vesicles can be delivered using various routes of administration (oral/intranasal, intramuscular, subcutaneous, intraperitoneal, and intradermal) (Gnopo et al., 2017). The ideal immunization route is dependent on the pathogen and bacterial component one is trying to elicit immune responses against. To elicit a mucosal immune response, one would likely use an oral or intranasal route whereas an intramuscular injection would result in a systemic immune response emanating from the draining lymph node nearest to the site of injection. A recent study demonstrated that an OMV (*N. meningitidis* derived) expressing the spike protein of SARS-CoV2 (OMV-mC-Spike) was able to elicit a strong and protective immune response when delivered intranasally to Syrian hamsters. These hamsters had a lower viral load in their throat as compared to mice that received OMV-mC-Spike intramuscularly (van der Ley et al., 2021). Additionally, BALB/c mice were injected with the OMV-mC-Spike either intranasally or intramuscularly and immunogenicity was assessed. Both routes of administration resulted in high serum IgG titers, but only the intranasally injected animals produced significant IgA responses as measured in the serum, nasal lavage, and lung (van der Ley et al., 2021). SARS-CoV2 infects through the mucosal surface and these data suggest intranasal immunization with a protective vaccine (OMV-mC-Spike) not only elicits sufficient systemic immunity, but also enhances localized mucosal immune responses (IgA).

## OMVs as vaccines

Outer membrane vesicles are naturally occurring bacterial delivery systems that can transfer cargo, such as nucleic acids, proteins, and lipids to proximal cells (or biofilms). The obvious application of OMVs is to use them to present antigens for vaccination. As outlined below, this method was first developed for vaccination against *N. meningitidis* serogroup B. Subsequently, OMVs have been derived from various bacterial strains with the intent of exploiting them for antigen delivery from homo- or heterologous strains. As mentioned earlier, it has been possible to express non-bacterial antigens and have them presented on the OMV surface following fusion to a carrier protein.

### Licensed vaccines

There have been two licensed OMV vaccines, VA-MENGOC-BC™ and Bexsero™, both protective against the invasive *N. meningitidis* serogroup B strain (MenB). While vaccines against multiple meningococcal serogroups of *Neisseria* have long existed, it had been challenging to make a vaccine against MenB because the major antigen expressed by this bacterium, PorA, is highly variable between strains. Traditional vaccine approaches have not been fruitful due to the low immunogenicity of PorA and the homology of this antigen to fetal neural tissue. Since OMVs can be made from the exact strain causing a meningitis outbreak, this approach has been successful in situations where there has been a clonal outbreak (Petousis-Harris, 2018).

VA-MENGOC-BC™ was the first licensed OMV vaccine and was approved for use in Cuba in 1987 following years of high rates of disease from a single serotype of MenB (B4:P1.15). This vaccine also included serogroup C and vaccine efficacy was estimated at 85% (Schwechheimer and Kuehn, 2015; Bottero et al., 2016). OMV vaccines were subsequently used in MenB outbreaks in Norway, New Zealand, and France. The vaccine used in the Norwegian and French (MenBVac) outbreaks displayed a quick decline in immune response over time, but surprisingly it also elicited broader protection against other Neisserial strains (Petousis-Harris, 2018; Balhuizen et al., 2021). The New Zealand epidemic of MenB peaked in 2001 and between 2004–2006 there was a mass immunization campaign with the OMV vaccine MeNZB™ and this vaccine was estimated to be 75% effective (Arnold et al., 2011; Petousis-Harris, 2018).

There is currently only one OMV-based vaccine licensed by the FDA and EMA, Bexsero™, produced by Glaxo Smith Kline (Brentford, United Kingdom). It is given intramuscularly to children and young adults aged 10–25 years old. This vaccine utilizes the MeNZB™ OMV vaccine administered during the New Zealand outbreak and it is formulated with three additional recombinant proteins (rMenB) that were identified by reverse vaccinology. This vaccine is estimated to be protective against 66–91% of the MenB strains worldwide (Petousis-Harris, 2018; Micoli and MacLennan, 2020). Interestingly, Bexsero™ has been found to be cross protective against *N. gonorrhoeae* (Semchenko et al., 2019).

### Bacterial vaccines in discovery

Many OMV vaccines are under development around the world. As OMVs can often be derived from the parent strain of a gram-negative pathogen, these types of vaccines are the least challenging to produce because the antigens needed are naturally expressed in the outer membrane in their native conformation, yet as mentioned earlier, such OMV vaccines would likely need manipulation to decrease endotoxin levels and increase vesiculation.

Following the successes with *N. meningitidis*, researchers are trying to develop a vaccine against the increasingly drug-resistant pathogen *N. gonorrhoeae*, a bacterium that has similar challenges as it evades immunity by antigenic variation, particularly of its pilin gene (Voter et al., 2020). Some of the other bacterial pathogens that have OMV vaccines in advanced discovery are the enteric pathogens *Shigella* spp., *Salmonella* spp., extraintestinal pathogenic *E. coli* (EXPEC), and *V. cholerae*. There are also efforts to develop OMV vaccines against *Mycobacterium tuberculosis* and non-typeable *H. influenzae* (Micoli and MacLennan, 2020). There are additional bacterial pathogens being explored to develop OMV-based vaccines in earlier stages of discovery research.

### Non-bacterial OMV based vaccines

As mentioned earlier in this manuscript, OMVs can be used to vaccinate against non-bacterial antigens. The examples provided herein were the use of influenza M2e antigen and the SARS-CoV2 spike protein, both of which mounted robust immune responses (Rappazzo et al., 2016; van der Ley et al., 2021). It has been reported by one group that they have constructed an OMV vaccine protective against both influenza and Middle East Respiratory Syndrome (MERS) viruses. The H1 hemagglutinin antigen of influenza and the receptor binding domain (RBD) of MERS were fused together and this plasmid was introduced into an *E. coli* strain. The resulting OMVs displayed the chimeric fusion protein and when mice were immunized with this OMV (prime and boost), they produced neutralizing antibodies to MERS RBD and hemagglutinin specific antibodies (Shehata et al., 2019).

Outer membrane vesicle vaccines have utility beyond fighting pathogens. A therapeutic cancer vaccine could potentially inhibit tumor growth or metastasis and destroy cancer cells present in the body after treatment. This could be accomplished by expressing non-endogenous protein on the outer membrane surface *via* carrier protein co-expression. One group produced a tumor-targeted OMV (HER2 antigen) carrying a therapeutic siRNA [fluorescently labeled kinesin spindle protein (KSP)]. After demonstrating proof of concept in various HER2-expressing *in vitro* cell lines, the Affi_HER2_OMV^siRNA^ was infused into a tumor xenograft murine model and after multiple injections and a number of days, it was found the group of mice receiving the targeted drug-loaded OMV displayed tumor growth inhibition of 66% as compared to the vehicle control (Gujrati et al., 2014).

A recent manuscript described a heterologous cancer OMV construct in which tumor antigens were fused to the ClyA carrier protein to demonstrate T cell driven anti-tumor immunity. As an alternative approach, they fused protein catcher systems [SpyCatcher (SpC) or SnoopCatcher (SnC)] to ClyA to enable the capture of various tumor neoantigens on the OMV outer membrane. These systems displayed on the outer membrane of the OMV can “catch” either a SpyTag or SnoopTag labeled protein through spontaneous isopeptide formation. The authors of that study successfully displayed multiple tumor antigens on the same OMV. This type of system provides flexibility in the neoantigens displayed on the OMV surface and could enable personalized treatment for the cancer patient (Cheng et al., 2021).

## Discussion

Outer membrane vesicles are natural nanoscale delivery systems and multiple groups are creatively engineering them to increase their utility. Their inherent immunogenicity provides adjuvant properties which makes them ideal candidates for vaccine development. Some of the challenges of this system revolve around consistency of yields, immunogenicity, toxicity, and loading. Efforts are currently underway to overcome these challenges. There has been discussion around building synthetic OMVs constructed from lipids combined with the antigens of interest, similar to LNP formulation. This could increase reproducibility of protein expression, decrease toxicity, and increase yields.

Fusion of antigens to an outer membrane protein allow for expression of non-native antigens on the OMV surface. This heterologous protein display opens up many possibilities as to how this platform could be used. Incorporation of targeting moieties (through genetic or chemical modification) can potentially allow for targeted drug delivery. While electroporation has been used to load cargo into the OMV, there may be more efficient ways to do this. Protein catcher systems displayed on the outer membrane make OMVs modular, allowing attachment of many different types of antigens. In conclusion, OMVs are a promising platform for vaccines and potentially for immunotherapy and drug delivery.

## Author contributions

LL wrote the manuscript and approved the submitted version.
